# Albiflorin Promotes Osteoblast Differentiation and Healing of Rat Femoral Fractures Through Enhancing BMP-2/Smad and Wnt/β-Catenin Signaling

**DOI:** 10.3389/fphar.2021.690113

**Published:** 2021-07-16

**Authors:** Jae-Hyun Kim, Minsun Kim, SooYeon Hong, Eun-Young Kim, Hyangsook Lee, Hyuk-Sang Jung, Youngjoo Sohn

**Affiliations:** Department of Anatomy, College of Korean Medicine, Kyung Hee University, Seoul, South Korea

**Keywords:** Paeonia lactiflora, albiflorin, osteoblast, bone morphogenetic protein 2, runt-related transcription factor 2, Wnt, femoral fracture

## Abstract

Fracture healing is related to osteogenic differentiation and mineralization. Recently, due to the unwanted side effects and clinical limitations of existing treatments, various natural product-based chemical studies have been actively conducted. Albiflorin is a major ingredient in *Paeonia lactiflora*, and this study investigated its ability to promote osteogenic differentiation and fracture healing. To demonstrate the effects of albiflorin on osteoblast differentiation and calcified nodules, alizarin red S staining and von Kossa staining were used in MC3T3-E1 cells. In addition, BMP-2/Smad and Wnt/β-catenin mechanisms known as osteoblast differentiation mechanisms were analyzed through RT-PCR and western blot. To investigate the effects of albiflorin on fracture healing, fractures were induced using a chainsaw in the femur of Sprague Dawley rats, and then albiflorin was intraperitoneally administered. After 1, 2, and 3 weeks, bone microstructure was analyzed using micro-CT. In addition, histological analysis was performed by staining the fractured tissue, and the expression of osteogenic markers in serum was measured. The results demonstrated that albiflorin promoted osteoblastogenesis and the expression of RUNX2 by activating BMP-2/Smad and Wnt/β-catenin signaling in MC3T3-E1 cells. In addition, albiflorin upregulated the expression of various osteogenic genes, such as alkaline phosphatase, OCN, bone sialoprotein, OPN, and OSN. In the femur fracture model, micro-CT analysis showed that albiflorin played a positive role in the formation of callus in the early stage of fracture recovery, and histological examination proved to induce the expression of osteogenic genes in femur tissue. In addition, the expression of bone-related genes in serum was also increased. This suggests that albiflorin promotes osteogenesis, bone calcification and bone formation, thereby promoting the healing of fractures in rats.

## Introduction

Bones are important structures that protect vital organs in the body and enable movement (Buckwalter and Cooper, 1987). Fracture refers to the continuity of bones being completely or incompletely broken by external forces. Delayed fracture recovery causes nonunion (Hak et al., 2014), which is a serious threat to the patient’s quality of life (Madureira et al., 2012). To treat fractures, a plaster cast is necessary, which limits the movement of the fracture site, and medication is administered in parallel (Jain, 2011). Various medications, such as bisphosphonates, parathyroid hormone (PTH 1–34), calcitonin and selective estrogen receptor modulators (SERMs), have a good effect in the treatment of fractures (Schatzker et al., 1979; Spiro et al., 2013; Xue et al., 2014; Hong et al., 2019), but they are not suitable for long-term treatment because they cause unwanted side effects, such as osteosarcoma, hypertension, syncope and hypercalcemia (Kennel and Drake, 2009; An, 2016; Minisola et al., 2019). In addition, some treatments are too expensive, and have several clinical problems, such as the complexity of the administration method (Borgstrom et al., 2010). To solve these problems, the discovery of natural product-based drugs that can treat fractures is a hot research topic.

Osteoblasts are well known to play a crucial role in bone metabolism (Shahi et al., 2017) and differentiate from mesenchymal stromal cells under appropriate stimulation (Grove et al., 2004). Injured bone area stimulates the recruitment, formation and activity of mesenchymal stem cells to form new bone (Wang et al., 2013). The main mechanism regulating osteoblastogenesis and bone mineralization is the bone morphogenetic protein 2 (BMP-2)/Smad pathway (Liu et al., 2018). BMP-2 phosphorylates Smad and translocates it into the nucleus, and then these signaling pathways upregulate the expression of Runt-related transcription factor 2 (RUNX2, also known as Cbfa1/PebpaA/AML), a key transcription factor for osteoblast differentiation (Jang et al., 2012). Another main mechanism of osteogenesis is the Wnt/β-catenin canonical pathway, which controls the proteasomal degradation of the transcription factor β-catenin. The β-catenin translocated to the nucleus binds T-cell factor/lymphoid enhancer factor (TCF/LEF) to activate the RUNX2 promoter (Yavropoulou and Yovos, 2007), and finally, these two pathways express osteogenic proteins such as osteocalcin (OCN), alkaline phosphatase (ALP), and bone sialoprotein (BSP) (Komori, 2010).

In traditional Korean medicine theory, fractures were thought to impair blood flow because of damage to bones and surrounding tissues, which was considered to be the main cause of the delayed healing rate of fractures. For this reason, to normalize blood circulation in the early stage of fracture, “Hwal-hyeol-yak; 活血藥”, which consists of herbs for invigorating blood and dispelling blood stagnation, was prescribed. After the blood flow was restored to normal, “Bo-hyeol-yak; 補血藥”, a medicine that replenishes the blood, was prescribed to treat damaged bones and tissues at the fracture site (Jin, 1985). In recent studies, the importance of angiogenesis has been demonstrated in the treatment of fractures using transgenic mice (Street et al., 2002; Wang et al., 2007). New blood vessels supply nutrients and oxygen and activate the regeneration of callus. They also provide a route for cartilage, bone forming cells and inflammatory cells to move to the fracture site. Based on this similarity, we anticipate that natural herbs that were traditionally used to improve blood flow could be used to treat fractures.

*Paeonia lactiflora* (PL), a representative type of “Bo-hyeol-yak”, has been used for various diseases, such as menstrual irregularities and menstrual pain, that are known to be caused by a lack of blood (Herbology, 2004) in East Asia, such as in Korea, China, and Japan, for more than 1,200 years. Previous studies have demonstrated that PL exerts various biological activities, such as anti-inflammatory effects (Ma et al., 2018), anti-cachexia effects in cancer patients (Bae et al., 2020), anti-ulcerative colitisand effect (Zhang et al., 2019), anti-skin-depigmenting effect (Qiu et al., 2016) and anti-migraine effects (Liao et al., 2019) Albiflorin, a monoterpene glycoside, is an active ingredient of PL. Albiflorin has also been found to exert pharmacological effects in diseases, such as improving lung inflammation (Cai et al., 2019), improving memory deficiency (Xu et al., 2019), preventing osteoporosis through antioxidant effects (Suh et al., 2013), and promoting mineralization (Yen et al., 2007). However, the mechanism and role of albiflorin and PL in fracture healing have not been demonstrated.

This study examined the effects of PL on osteoblast differentiation and fracture healing and then researched the osteoblast-promoting effects of albiflorin, which are known as the active ingredients of PL. Finally, we explored the mechanism by which albiflorin enhances osteoblastogenesis using osteoblast-like cells and suggested the possibility of a new fracture treatment alternative in femoral fractured rats.

## Materials and Methods

### Reagents

Albiflorin (C_23_H_28_O_11_) and paeoniflorin (C_23_H_28_O_11_) isolated from *Paeonia lactiflora* (HPLC purity>98%) were purchased from Wuhan ChemFaces Biochemical Co., Ltd. (Hubei, China). Minimum essential Eagle’s medium, α-modification (α-MEM), fetal bovine serum (FBS), penicillin/streptomycin (P/S), Dulbecco’s phosphate-buffered saline (DPBS) and normal serum were supplied by Gibco (Waltham, MA, United States). Ascorbic acid, beta-glycerophosphate, Noggin (BMP-2 inhibitor; cat no: SRP3227), protease inhibitor, phosphatase inhibitor 2, 3 and CCK-8 solution [WST-8; 2-(2-methoxy-4-nitrophenyl)-3-(4-nitrophenyl)-5-(2,4-disulfophenyl)-2H-tetrazolium, monosodium salt] were purchased from Sigma-Aldrich; Merck KGaA (Darmstadt, Germany). Alizarin Red S was supplied by Duksan Co., Ltd. (Seoul, Korea). KYA1797K (Wnt/β-catenin inhibitor; cat no: ab229170), anti-bone morphogenetic protein 2 (BMP-2; cat no: ab14933), anti-runt-related transcription factor 2 (RUNX2; cat no: ab76956), anti-osteocalcin (OCN; cat no: ab13418), anti-Sp7/Osterix (Osterix; cat no: ab209484) and anti-SMAD1/5/9 (SMAD1/5/9; cat no: ab80255) were purchased from Abcam (Cambridge, MA, United States). Anti-phospho-SMAD1/5 (p-SMAD1/5; cat no: 9516S) was shipped by Cell Signaling Technology, Inc (Danvers, MA, United States). Anti-actin (Actin; cat no: sc-8432) was purchased from Santa Cruz Biotechnology, Inc (Dallas, TX, United States). Nitrocellulose membranes were supplied by Bio-Rad, Laboratories, Inc. (Hercules, CA, United States). Horseradish peroxidase (HRP)-conjugated secondary antibody was supplied by Jackson ImmunoResearch Laboratories, Inc (West Grove, PA, United States). TRIzol was purchased from Takara Bio, Inc. (Otsu, Japan). A Reverse Transcription Kit was supplied by Invitrogen (Waltham, MA, United States). A Polymerase Chain Reaction Kit was purchased from Kapa Biosystems (Indianapolis, IN, United States). Proteinase K was supplied by Thermo Fisher Scientific (Waltham, MA, United States). Biotinylated secondary antibody, ABC HRP Kit (Peroxidase, Standard) and 3,3′-diaminobenzidine (DAB) solution were purchased from Vector Labs (Burlingame, CA, United States). All of the reagents used in this study were of cell culture grade.

### Preparation of Paeonia Lactiflora

PL was purchased from Omniherb (Seoul, Korea). PL was verified by Professor Yungmin Bu of the Herbology Laboratory at Kyung Hee University. The voucher specimens in this study are kept in the department of anatomy herbarium of Korean medicine of Kyung Hee University (ref. no. KHU-ANA-Et004). PL was precipitated in 80% ethanol for 1 week. Then, the extract was filtered using filter paper and lyophilized to obtain a powder (yield: 5.9%). For *in vitro* experiments, the powder was dissolved in dimethylsulfoxide (DMSO) and then filtered through a syringe filter (pore size, 0.22 µm). DMSO did not exceed 0.1% of the total volume of the cell medium.

### Cell Culture

MC3T3-E1 osteoblast precursor cells were obtained from American Type Culture Collection (ATCC-CRL-2593, Manassas, VA, United States). MC3T3-E1 cells were cultured in α-MEM (without ascorbic acid) supplemented with 10% FBS and 1% penicillin-streptomycin. Cells were cultured in a cell incubator maintained at 37°C and 5% CO_2_. MC3T3-E1 cells were differentiated into osteoblasts in osteogenic medium (α-MEM supplemented with 25 μg/ml ascorbic acid and 10 mM β-glycerophosphate).

### Cell Viability Assay

To analyze cell cytotoxicity, MC3T3-E1 cells were cultured with or without albiflorin and PL in 96-well plates (1 × 10^4^ cells) for 1–3 days, and then 10 µl of CCK-8 solution was added to each well. After 2 h of incubation, the plate was measured at 490 nm using an enzyme-linked immunosorbent assay reader (Versamax; Molecular Devices, LLC, Sunnyvale, CA, United States). The percentage of cell viability was calculated based on non-treated MC3T3-E1 cells.

### Bone Mineralization Analysis and Alkaline Phosphatase Staining

MC3T3-E1 cells were cultured in 24-well plates (1 × 10^4^ cells) and incubated in osteogenic medium with or without albiflorin, paeoniflorin and PL for 14 days. The culture medium was changed to fresh medium of the same type every 2 days. Then, the culture medium was removed and washed twice with DPBS. The cells were fixed with 80% ethanol at 4°C for 1 h. For alizarin red S staining, calcified nodules were stained with alizarin red S solution for 5 min. The stained dye was extracted with 10 mM sodium phosphate (pH 7.0) containing 10% cetylpyridinium chloride for 15 min, and absorbance was measured at a wavelength of 570 nm using an enzyme-linked immunosorbent assay reader. For von Kossa staining, calcified nodules were fixed with 80% ethanol and washed with deionized water. Subsequently, 1% silver nitrate was added to the plate and reacted for 40 min in UV. The stained plate was reacted with 5% sodium thiosulfate for 10 min, rinsed with deionized water (DW) and dried. The plate was photographed with a camera. ALP was stained with the AP Blue Staining Kit (Systembio, Palo Alto, CA, United States) according to the manufacturer’s protocol. The plate was photographed with an inverted microscope.

### Western Blot Analysis

MC3T3-E1 cells were cultured in 60π dishes (5 × 10^5^ cells) and incubated in osteogenic medium with or without albiflorin and PL for 2 days. The cells were washed 3 times using DPBS and then dissolved in RIPA buffer (50 mM Tris-Cl, 150 mM NaCl, 1% NP-40, 0.5% sodium deoxycholate and 0.1% SDS with protease inhibitor and phosphatase inhibitor 2 and 3 cocktails) to extract the protein. Equal amounts of protein (30 µg) were separated by 10% sodium dodecyl sulfate-polyacrylamide gel electrophoresis and electrotransferred onto nitrocellulose membranes. Nonspecific proteins were blocked with 5% skim milk for 1 h at room temperature, and primary antibody was diluted in 1% BSA and reacted overnight at 4°C (dilution, 1:1,000). Then, the HRP-conjugated secondary antibody was reacted at room temperature for 1 h, and protein expression was measured using enhanced chemiluminescence (ECL) solution (GE Healthcare Life Science, Chicago, IL, United States). The density of the band was quantified using ImageJ software (National Institutes of Health, Bethesda, MD, United States). The expression of proteins was normalized to that of actin.

### Reverse Transcription-Polymerase Chain Eeaction Analysis

MC3T3-E1 cells were cultured in 6-well plates (2 × 10^5^ cells) and incubated in osteogenic medium with or without albiflorin and PL for 4 days. Total RNA was extracted using TRIzol according to the manufacturer’s protocol. Two micrograms of total RNA were synthesized with complementary deoxyribonucleic acid (cDNA) using reverse transcriptase. The cDNA was subjected to RT-PCR using Taq polymerase and a C1000 Touch™ Thermal Cycler. The mouse primers used were as Table 1. The PCR products were electrophoresed on a 1% agarose gel stained with SYBR green (Invitrogen). The expression of target genes was normalized to that of GAPDH.

**TABLE 1:** Sequences of primers used for reverse transcription PCR.

Gene name	Sequence	Base pairs	Annealing Tm (˚C)	Cycles	References
mBMP-2 (*Bmp2*)	F: GGG​ACC​CGC​TGT​CTT​CTA​GT	162	57	40	NM_007553.3
	R: TCA​ACT​CAA​ATT​CGC​TGA​GGA				
mRUNX2 (*Runx2*)	F: CGGCCCTCCCTGAACTCT	75	60	38	NM_001145920.2
	R: TGC​CTG​CCT​GGG​ATC​TGT​A				
mALP (*Alpl*)	F: CGG​GAC​TGG​TAC​TCG​GAT​AA	208	55	42	NM_001287172.1
	R: TGA​GAT​CCA​GGC​CAT​CTA​GC				
mOCN (*Bglap*)	F: GCA​ATA​AGG​TAG​TGA​ACA​GAC​TCC	147	59	45	NM_001032298.3
	R: GTT​TGT​AGG​CGG​TCT​TCA​AGC				
mBSP (*Ibsp*)	F: AAA​GTG​AAG​GAA​AGC​GAC​GA	215	53	35	NM_008318.3
	R: GTT​CCT​TCT​GCA​CCT​GCT​TC				
mWnt10b (*Wnt10b*)	F: TTC​TCT​CGG​GAT​TTC​TTG​GAT​TC	118	59	42	NM_011718.2
	R: TGC​ACT​TCC​GCT​TCA​GGT​TTT​C				
mβ-catenin (*Ctnnb1*)	F: TTC​TCT​CGG​GAT​TTC​TTG​GAT​TC	158	59	41	NM_001165902.1
	R: TGC​ACT​TCC​GCT​TCA​GGT​TTT​C				
mLRP5 (*Lrp5*)	F: AAG​GGT​GCT​GTG​TAC​TGG​AC	220	58	40	Kim et al. (2014)
	R: AGA​AGA​GAA​CCT​TAC​GGG​ACG				
mLRP6 (*Lrp6*)	F: TTC​TCT​CGG​GAT​TTC​TTG​GAT​TC	227	58	40	Zhai et al. (2016)
	R: TGC​ACT​TCC​GCT​TCA​GGT​TTT​C				
mDVL2 (*Dvl2*)	F: GCT​TCC​ACA​TGG​CCA​TGG​GC	195	64	40	Kim et al. (2014)
	R: TGG​CAC​TGC​TGG​TGA​GAG​TCA​CAG				
mCcnd1 (*Ccnd1*)	F: GAAGGAGATTGTGCCATC	141	55	40	Zhai et al. (2016)
	R: TTCTTCAAGGGCTCCAGG				
mOSN (*Sparc*)	F: AAA​CAT​GGC​AAG​GTG​TGT​GA	217	54	25	NM_001290817.1
	R: TGC​ATG​GTC​CGA​TGT​AGT​C				
mOPN (*Spp1*)	F: TCT​GAT​GAG​ACC​GTC​ACT​GC	170	53	33	NM_001290377.1
	R: AGG​TCC​TCA​TCT​GTG​GCA​TC				
mGAPDH (*Gapdhs*)	F: ACT​TTG​TCA​AGC​TCA​TTT​CC	267	58	30	NM_008084.3
	R: TGC​AGC​GAA​CTT​TAT​TGA​TG				
rBMP-2 (*Bmp2*)	F: GAA​GCC​AGG​TGT​CTC​CAA​GAG	122	58	33	NM_017178.1
	R: GTG​GAT​GTC​CTT​TAC​CGT​CGT				
rRUNX2 (*Runx2*)	F: ATC​CAG​CCA​CCT​TCA​CTT​ACA​CC	199	58	33	NM_053470.2
	R: GGG​ACC​ATT​GGG​AAC​TGA​TAG​G				
rGAPDH (*Gapdhs*)	F: CCT​GCA​CCA​CCA​ACT​GCT​TA	120	58	31	NM_017008.4
	R: GGC​CAT​CCA​CAG​TCT​TCT​GAG				

mBMP-2, mouse bone morphogenetic protein 2; mRUNX2, mouse runt-related transcription factor 2; mALP, mouse alkaline phosphatase; mOCN, mouse osteocalcin; mBSP, mouse bone sialoprotein; mWnt10b, mouse Wnt10b; mβ-catenin, mouse β-catenin; mLRP5, mouse low-density lipoprotein receptor-related protein 5; mLRP6, mouse low-density lipoprotein receptor-related protein 6; mDVL2, mouse segment polarity protein dishevelled homolog DVL-2; mCcnd1, mouse cyclin D1; mOSN, mouse osteonectin; mOPN; mouse osteopontin; mGAPDH, mouse glyceraldehyde 3-phosphate dehydrogenase; rBMP-2, rat BMP-2; rRUNX2, rat RUNX2; rGAPDH, rat GAPDH.

### High-Performance Lliquid Chromatography–Photodiode Array Assay for Paeonia Lactiflora

The HPLC (Waters Alliance 2695)-PDA (Waters 2996) detector system was used to analyze albiflorin and paeoniflorin, which are the major components of PL. An XBridge ™ C18 (5 mm × 250 mm; Waters, Milford, MA, United States) column was used for analysis, the injection force of the sample was 10 μl, and the flow rate was 0.8 ml/min. Acetonitrile and water (H_2_O) containing 1% acetic acid were used as the mobile phase, and the gradient solvent condition was 1:9. The detection wavelength of the chromatogram was 232 nm.

### Animal Fracture Model and Radiological Evaluation

The *in vivo* experiment of this study was carried out after obtaining the permission of the Kyung Hee University Animal Committee [PL: ref. no. KHUASP(SE)-16–115; Albiflorin: ref. no. KHUASP(SE)-19–271]. Eight-week-old male Sprague Dawley (SD) rats were purchased from Raon Biotech Co., Ltd (Seoul, Korea). Rats were given a one-week stabilization period to adapt to the environment. The rearing environment of the rats was maintained at 22–24°C and 55% humidity under a 12 h light/dark cycle with free access to feed and water. To induce femoral fracture, rats were anesthetized with isoflurane solution (3% end-tidal concentration). Depilation of the lateral right femur was performed, and an incision was made in the skin from the knee to the femoral head. The gluten superficialis and biceps femoris muscles were separated to expose the femur, and a chainsaw was used to cut the mid-shaft of the femur. Subsequently, a 1.1 mm K-wire was inserted into the bone marrow in the anterior direction through the knee in the osteotomy site (Auregan et al., 2013). After fracture surgery, antibiotics (gentamicin, 4 mg/kg) were intraperitoneally administered to prevent infection at the surgical site. No rats died due to femur fracture surgery. There were no wound infections or complications. The following standards were set for ending the animal experiment on humane grounds: 1) severe infection, laceration and bleeding at the surgical site; 2) difficulty taking food or water due to uncomfortable walking; 3) weight loss of 20% or more compared to the control group of the same age; 4) difficulty maintaining normal posture due to weakness; 5) unconsciousness without reaction to external stimulation.

Experimental 1, to determine the effect of PL on bone healing, twenty-four rats were divided into three groups (*n* = 8/each group) as follows: group 1 was sham operated and orally administered distilled water daily, group 2 was fracture-induced and orally administered distilled water daily, and group 3 was fracture-induced and orally administered 100 mg/kg PL daily. After 5 weeks of treatment, blood was collected through a heart puncture under deep breathing anesthesia, and then the rats were sacrificed by cervical dislocation. The femur was extracted, the wire was removed and fixed in 10% neutral buffered formalin (NBF) for 2 days after extraction.

**FIGURE 1:**
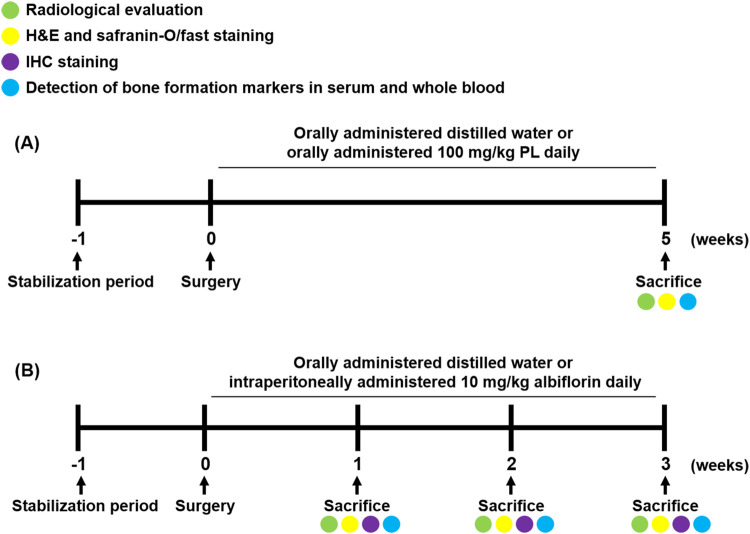
Schedule of fracture-induced animal experimental. **(A)** Experimental design to investigate the effect of PL on fracture recovery. **(B)** Experimental design to demonstrate the process of albiflorin on fracture healing.

Experimental 2, to determine the effect of albiflorin on the bone healing process, forty-eight rats were divided into nine groups (*n* = 6/each group) as follows: group 1 was sham operated and orally administered distilled water daily for 1 week, group 2 was fracture-induced and orally administered distilled water daily for 1 week, and group 3 was fracture-induced and intraperitoneally administered 10 mg/kg albiflorin daily for 1 week. Group 4 was sham operated and orally administered distilled water daily for 2 weeks, group 5 was fracture-induced and orally administered distilled water daily for 2 weeks, and group 6 was fracture-induced and intraperitoneally administered 10 mg/kg albiflorin daily for 2 weeks. Group 7 was sham operated and orally administered distilled water daily for 3 weeks, group 8 was fracture-induced and orally administered distilled water daily for 3 weeks, and group 9 was fracture-induced and intraperitoneally administered 10 mg/kg albiflorin daily for 3 weeks. The sacrifice and sampling were performed using the same methods as in the PL experiment. The animal experimental design conducted in this experiment is illustrated in Figure 1. The fractured femur was analyzed using micro-CT analysis (SkyScan1176, Skyscan, Kontich, Belgium) with a source set at 50 kV/200 μA, 8.9 μm isotropic resolution, and an aluminum filter of 0.5 mm. The analyzed femur was visualized using DataViewer software (Skyscan v.1.5.1.9). The microstructure of the callus was measured on 400 slides using Skyscan software (Skyscan v1.15.4.0) at the center of the cut site.

### Detection of Bone Formation Markers in Serum and Whole Blood

The blood was stored in an SST tube (BD Vacutainer, BD Biosciences, CA, USA) for over 30 min at room temperature. Then, serum was separated by centrifugation at 4,000 rpm, 4°C, and 20 min. The concentrations of ALP, AST and ALT in serum were detected by DKkorea Inc (Seoul, Korea). The concentration of OCN in serum was analyzed using the Osteocalcin ELISA Kit (cat: LS-F12230, LifeSpan BioSciences Inc., WA, United States). mRNA from whole blood was extracted using TRIzol as described above. Two micrograms of mRNA were synthesized with cDNA, and RT-PCR was performed using Taq polymerase and a C1000 Touch ™ Thermal Cycler. The rat primers used were as Table 1. The PCR products were electrophoresed on a 1% agarose gel stained with SYBR green. The expression of target genes was normalized to that of GAPDH.

### Hematoxylin and Eosin, Safranin-O/Fast green and Immunohistochemistry Staining

The fixed femur was decalcified for 8 weeks with 10% ethylenediaminetetraacetic acetic acid (ETDA)-2Na. Thereafter, the tissue was dehydrated with an ethanol series, cleared with xylene and embedded in paraffin. Then, the tissue was sliced into 5-µm-thick sections using a rotary microtome (Leica Biosystems, RM2125 RTS, Wetzlar, Germany). Morphological changes in the callus and cartilage area were detected by H and E staining and safranin O staining, respectively. To proceed with IHC staining, tissues were deparaffinized and then hydrated. The antigen was retrieved with proteinase K (Sigma-Aldrich; Merck KGaA) for 30 min at 60°C. The tissue was immersed in H_2_O_2_ for 15 min to regulate endogenous peroxidase activity at room temperature, and nonspecific protein was blocked with 10% normal serum for 30 min at 4°C. Primary antibodies were reacted overnight at 4°C (dilution, 1:200). The tissue was next washed with PBS and reacted with a biotinylated second antibody for 1 h at room temperature and then with ABC solution for 30 min. The expression was performed using DAB solution according to the manufacturer’s protocol. The dyed sample was photographed at 5 sections per tissue using a light microscope (BX51, Olympus, Shinjuku, Japan).

### Statistical Analysis

All experiments were conducted at least three times independently. All data are presented as the mean ± standard error of the mean (SEM). Analysis was performed using GraphPad Prism 5 software (version 5.01, Graphpad Softwatre Inc., San Diego, United States). The significance of differences between the groups was assessed by *t*-test or one-way analysis of variance (ANOVA), followed by Dunnett’s post hoc analysis. If the *p* value was less than 0.05, it was considered to be statistically significant.

## Results

### Paeonia Lactiflora Promoted Osteogenic Differentiation in MC3T3-E1 Cells by Enhancing Bone Morphogenetic Protein-2 Signaling

**FIGURE 2:**
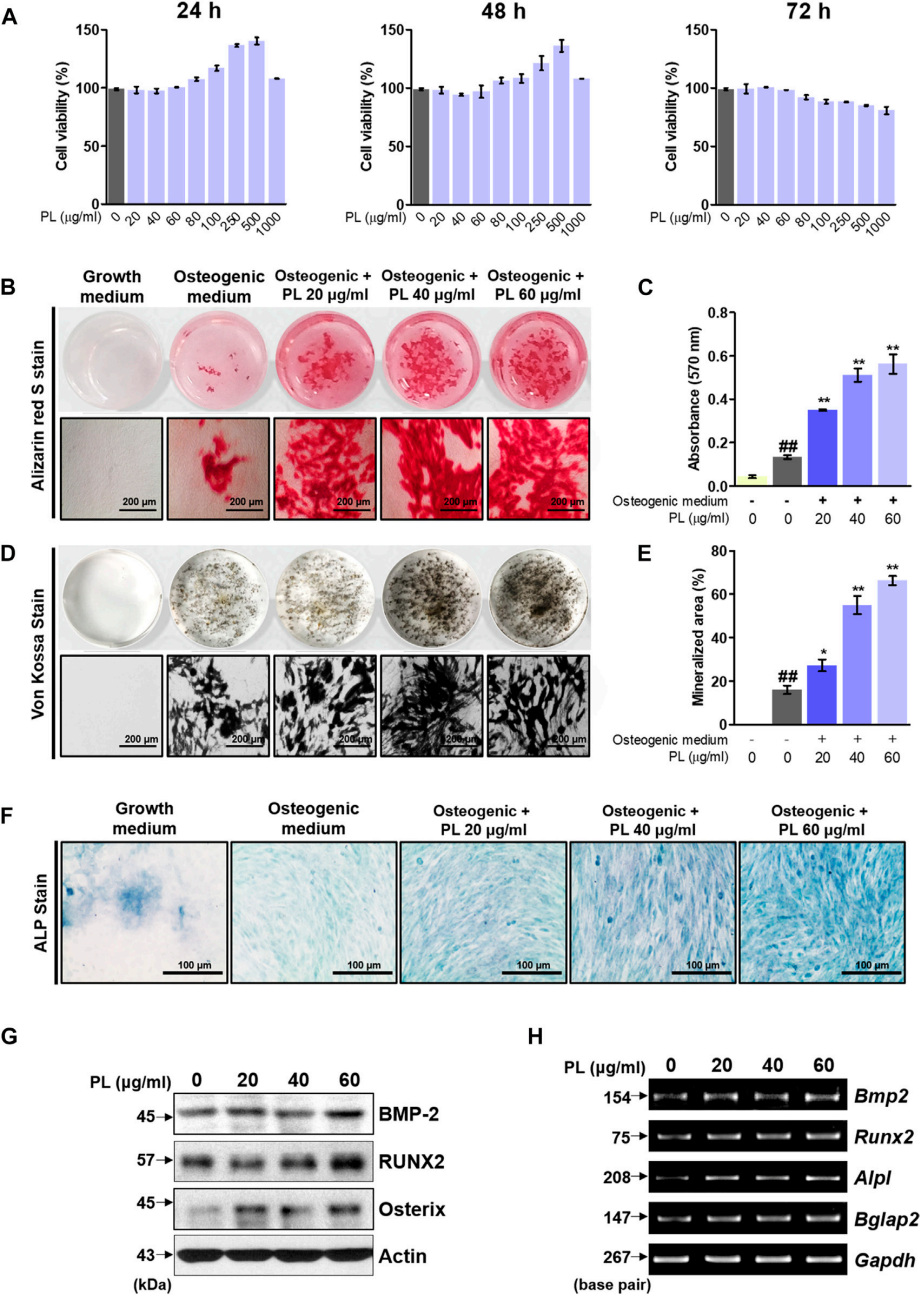
Effect of PL on osteoblast differentiation and bone mineralization. **(A)** The toxicity of PL in MC3T3-E1 cells was determined using a CCK-8 assay. **(B)** The effect of PL on mineralized nodule formation was analyzed by alizarin red S (×100 magnification, 200 μm). **(C)** The stained dye was extracted and measured at an absorbance of 570 nm by ELISA. **(D)** A plate view of von Kossa staining was captured using a camera and microscope (×100 magnification, 200 μm). **(E)** The mineralized area was measured using ImageJ software. **(F)** ALP expression in MC3T3-E1 cells was determined using an ALP Staining Kit (×200 magnification, 100 μm) **(G)** The protein levels of BMP-2, RUNX2 and Osterix were measured by western blot. **(H)** RNA expression of BMP-2 (*Bmp2*), RUNX2 (*Runx2*), ALP (*Alpl*) and OCN (*Bglap*) was detected by RT-PCR. These are presented as the mean ± SEM (*n* = 3 for each experimental). ^##^ *p* < 0.01 vs. the growth medium cells; **p < 0.01 and *p < 0.05 vs. osteogenic medium cells.

Before the *in vitro* experiment, CCK-8 was used to verify cytotoxicity to confirm the toxicity of PL. The CCK-8 method measures the amount of formazan produced, which is proportional to the number of living cells. It is very stable and used in many studies with low toxicity. Cell viability of less than 90% compared to that of non-treated cells was considered to indicate toxicity. Examining the effect of PL on cell viability showed that PL did not affect MC3T3-E1 cells at a concentration of 0–60 μg/ml in 72 h (Figure 2A). Thus, *in vitro* experiments were conducted at 60 μg/ml or less. Osteoblast differentiation and activity were measured using two methods: mineralized nodule formation and ALP staining. MC3T3-E1 cells were treated with osteogenic medium to differentiate osteoblasts. Mineralized nodules formed on the plate were stained with alizarin red S and von Kossa staining, and PL stimulated the formation of mineralized nodules in a concentration-dependent manner (Figures 2B,D). Quantitative analysis of alizarin red S dye and the von Kossa-stained mineralized area showed that PS stimulated osteoblast differentiation and activity in a concentration-dependent manner (Figures 2C,E). Moreover, the media containing PL dramatically increased the expression of ALP-positive cells (blue intensity and area in the plate) (Figure 2F). Next, the mechanism of the effect of PL on osteoblast activity at the protein and mRNA levels was verified. At the protein level, PL upregulated the expression of BMP-2, RUNX2, and Osterix (Figure 2G). It also induced the mRNA expression of osteoblastogenesis-related genes such as BMP-2, RUNX2, ALP, and OCN (Figure 2H).

### Effect of Paeonia Lactiflora on Femoral Fractured Rats

**FIGURE 3:**
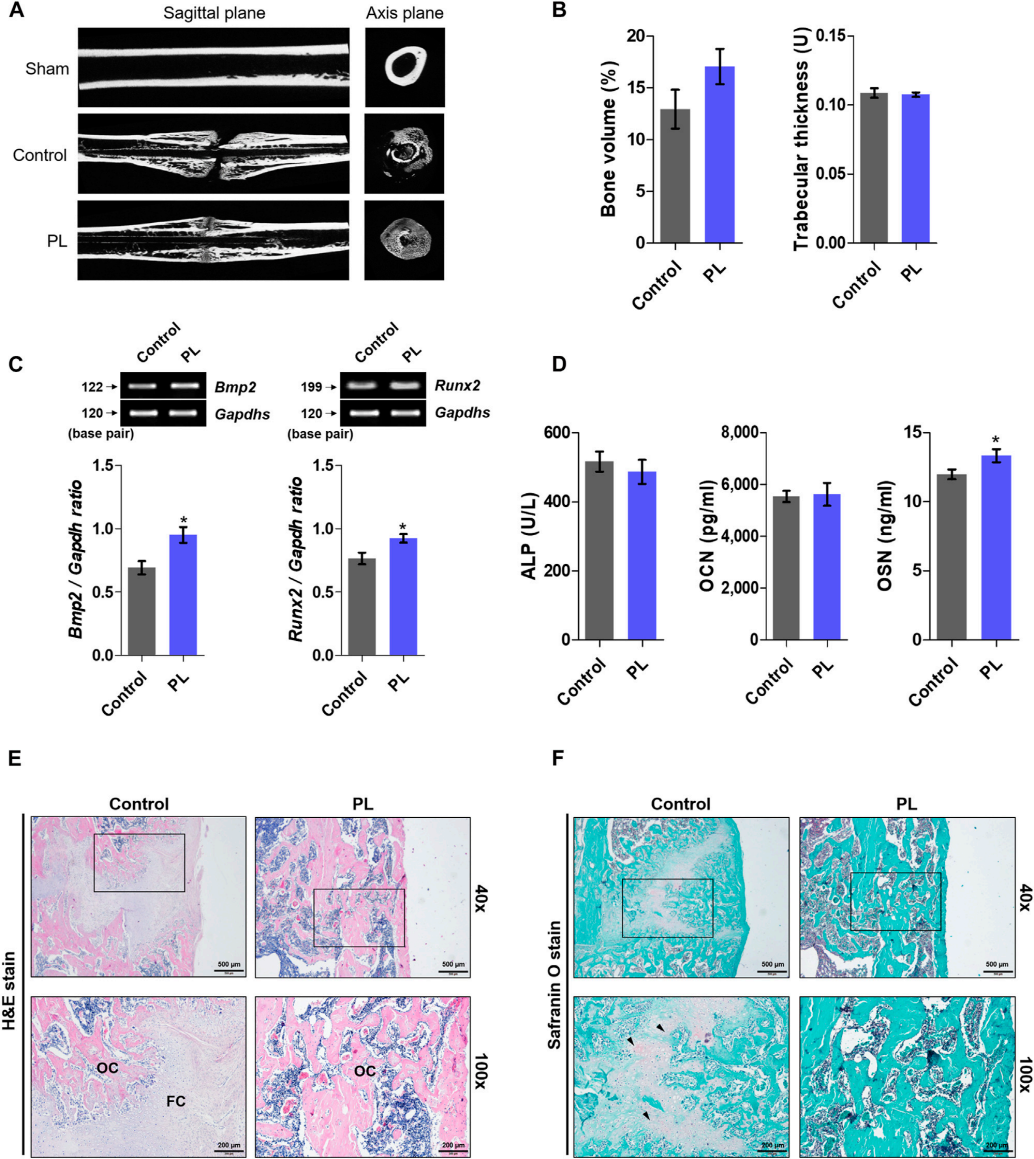
Effect of PL on healing of rat femoral fractures **(A)** Sagittal plane (upper panel) and axis plane (lower panel) micro-CT image of a fracture-induced femur at 5 weeks **(B)** Bone microstructure was analyzed by micro-CT. **(C)** mRNA expression of osteogenic markers such as BMP-2 and RUNX2 was measured in whole blood **(D)** The expression of ALP, OCN and OSN was measured in serum. Femoral tissue was embedded in paraffin, and histological analysis was performed. **(E)** Changes in fibrous and osseous callus at the fracture site were observed by H and E staining. The abbreviations in the H and E staining are as follows: OC, osseous callus; FC, fibrous callus (magnification: ×40 and 100×, scale bar: 500 and 200 µm) **(F)** Cartilage areas in the fractured sites were compared by safranin O staining (magnification: ×40 and 100×, scale bar: 500 and 200 µm). These results are presented as the mean ± SEM (*n* = 8 for each group). *p < 0.05 vs. control.

After obtaining promising *in vitro* results, we demonstrated the effect of PL on promoting fracture healing. As shown in Figures 3A,B, the femurs extracted from SD rats sacrificed after fracture induction were analyzed using micro-CT. The PL group showed a cleaner fracture line than the control group. In addition, the microstructure analysis showed that bone volume increased, but the difference was not significant, and there was no significant difference in trabecular thickness. As shown in Figures 3C,D, PL was confirmed to cause changes in osteogenic markers in whole blood and serum. mRNA expression of *Bmp2* and *Runx2* in whole blood showed a significant increase in the PL group. In addition, the concentration of OSN in serum was significantly increased. However, the PL group did not significantly affect the expression of ALP and OCN in serum. As shown in Figure 3E, five weeks after the induction of fracture, fibrous callus was still observed in the control group. In the PL group, only osseous callus was observed. The fracture line, collagen fibers, cartilage, and trabecular bone changes in the callus of fractured femurs were stained with safranin O (Figure 3F). The PL group had a smaller cartilage area than the control group, and the fracture line disappeared. These results are the same as the H and E results.

### Detection of the Active Ingredients of Paeonia Lactiflora and Verification of Their Osteoblast Differentiation-Promoting Ability

**FIGURE 4:**
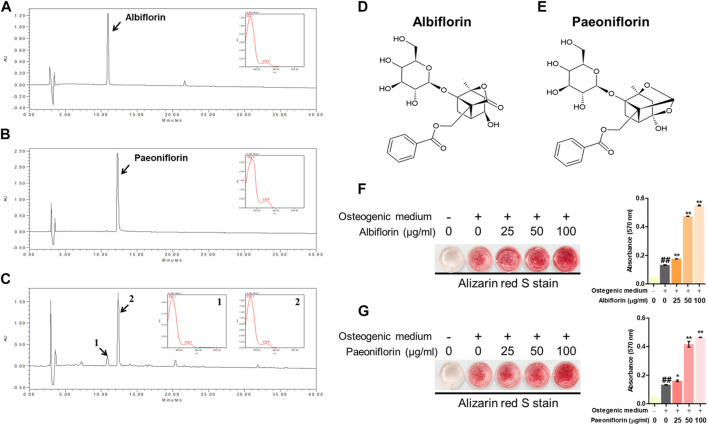
HPLC of. **(A)** albiflorin, **(B)** paeoniflorin, and. **(C)** PL. Each standard peak was detected at 232 nm. Molecular structure of **(D)** albiflorin and **(E)** paeoniflorin. **(F)** and **(G)** Alizarin red S staining was used to analyze the effects of albiflorin and paoniflorin on calcified nodule formation. These results are presented as the mean ± SEM (*n* = 3 for each experimental). ##*p* < 0.01 vs. growth medium cells; **p < 0.01, *p < 0.05 vs. osteogenic medium cells.

We identified which components in the PL affected osteoblast differentiation. Albiflorin and paeoniflorin are known as active ingredients of PL. The HPLC profiles obtained in this study are shown in Figures 4A,B. The retention times of albiflorin (10.937 min) and paeoniflorin (21.519 min) were determined in the chromatogram. In addition, PL ethanol extract was verified at the same retention time as albiflorin and paeoniflorin. (Figure 4C). The molecular structure of the ingredients is as follows (Figures 4D,E). Alizarin red S staining was performed to compare the effect of enhancing the osteoblast activity of the two components. Both albiflorin and paeoniflorin promoted the formation of calcified nodules in a dose-dependent manner (Figure 4F,G). However, the absorbance of the extracted dyes showed that albiflorin had higher osteoblast differentiation activity than paeoniflorin.

### Effect of Albiflorin on Osteoblast Differentiation and Bone Mineralization

**FIGURE 5:**
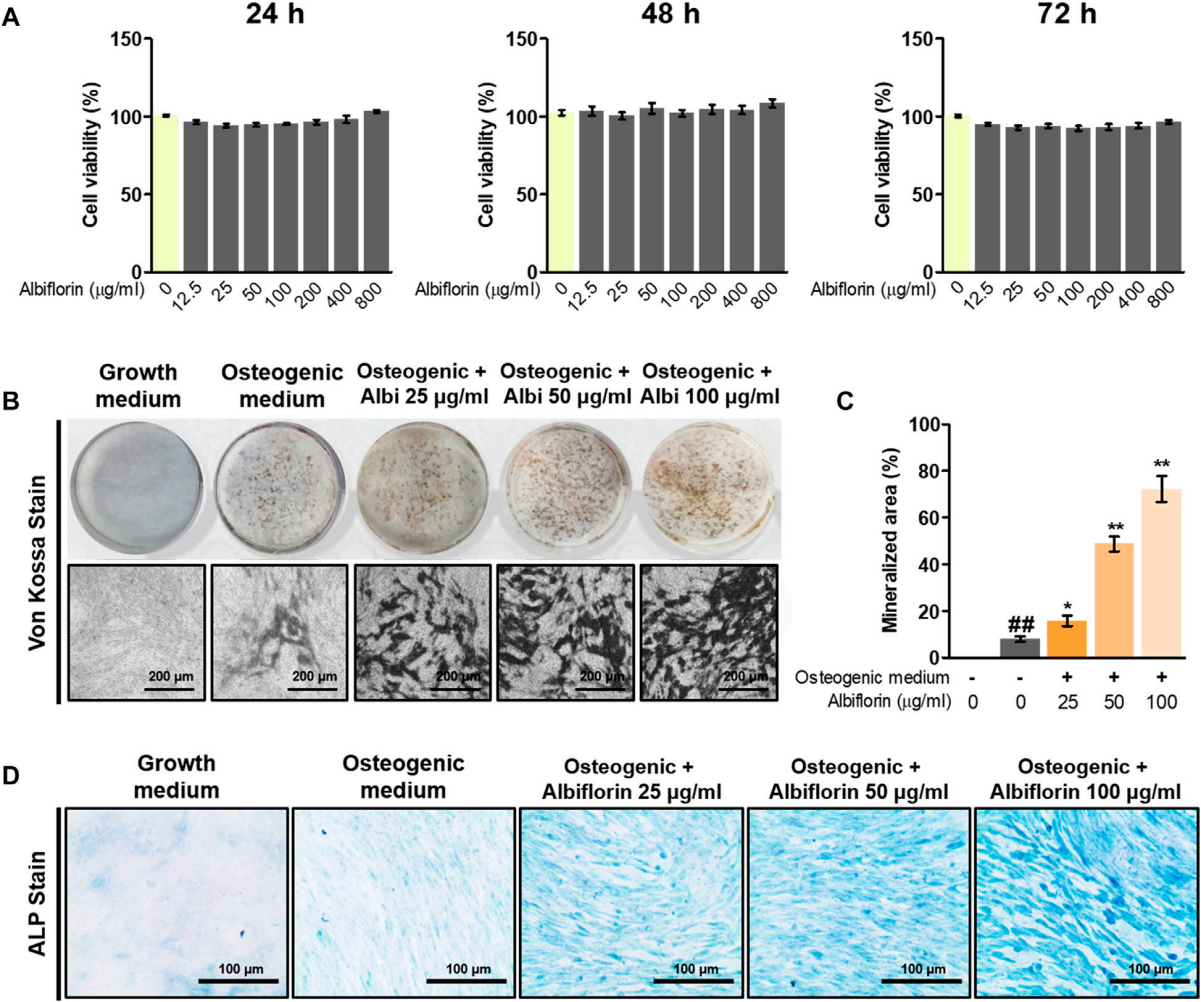
Effect of albiflorin on osteoblast differentiation and bone mineralization. **(A)** The toxicity of albiflorin in MC3T3-E1 cells was determined using a CCK-8 assay. **(B)** The effect of albiflorin on mineralized nodule formation was analyzed by von Kossa staining using a camera and microscope (×100 magnification, 200 μm). **(C)** The mineralized area was measured using ImageJ software. **(D)** ALP expression in MC3T3-E1 cells was determined using an ALP Staining Kit (×200 magnification, 100 μm). These results are presented as the mean ± SEM (*n* = 3 for each experimental). ##*p* < 0.01 vs. growth medium cells; **p < 0.01, *p < 0.05 vs. osteogenic medium cells.

The cytotoxicity of albiflorin was verified using CCK-8 methods, and albiflorin showed no cytotoxicity at all concentrations (12.5–800 μg/ml) in MC3T3-E1 cells (Figure 5A). Although cytotoxicity was not observed at any concentration, albiflorin at a concentration of 100 μg/ml had already shown sufficient ability to promote osteoblast differentiation, so the experiment was not conducted at concentrations above 200 μg/ml (data not shown). Differentiated osteoblasts were analyzed by Von Kossa staining. Albiflorin significantly increased the formation of calcified nodules (Figures 5B,C) and enhanced the area of ALP-positive cells in a concentration-dependent manner (Figure 5D).

### Albiflorin Increases the Expression of Bone Morphogenetic Protein-2/Smad and Wnt/β-Catenin Signaling

**FIGURE 6:**
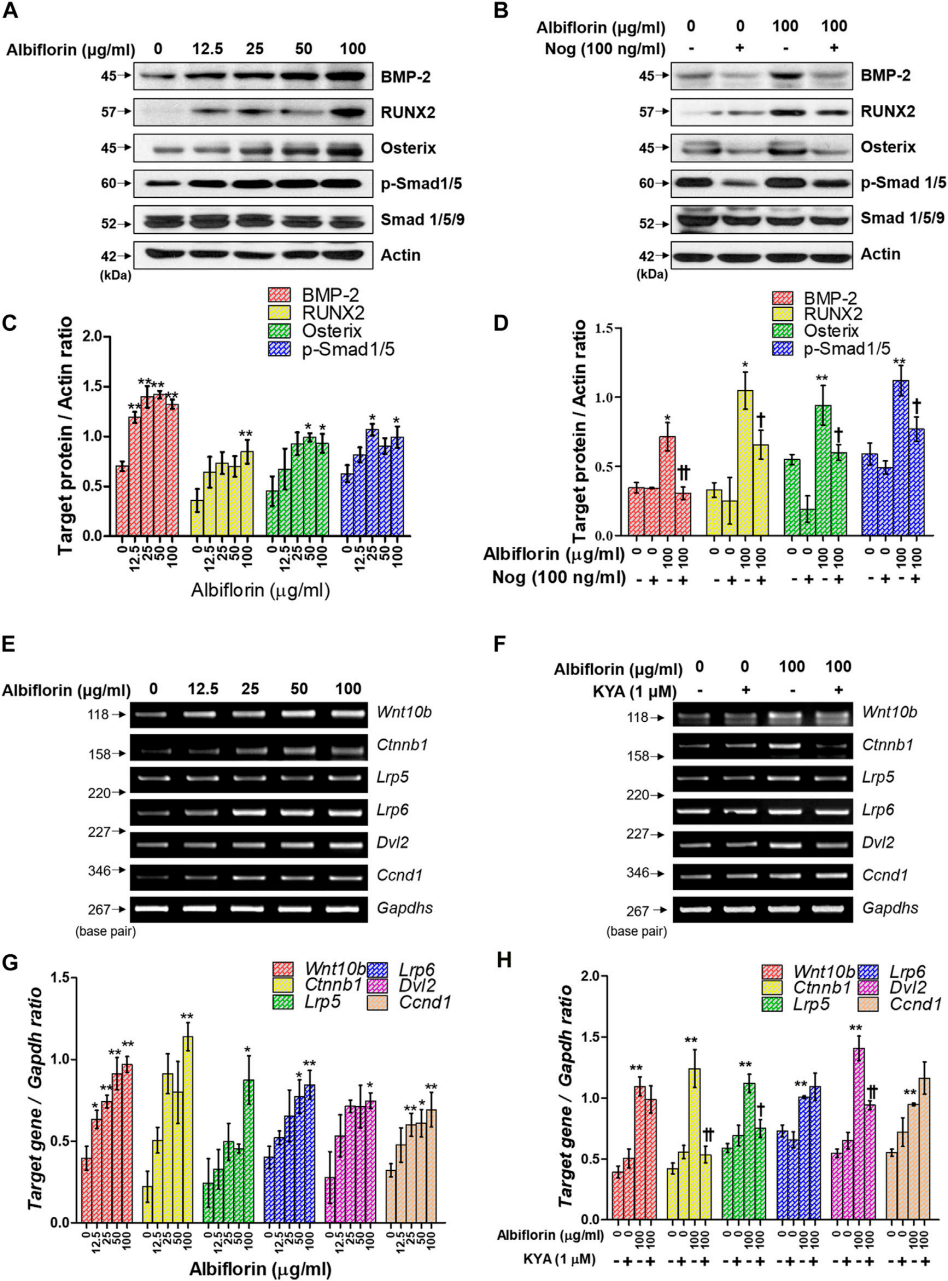
Effect of albiflorin on BMP-2/Smad and Wnt/β-catenin signaling **(A, B)** The effect of albiflorin on BMP-2 signaling-related protein expression was analyzed with or without the BMP-2 inhibitor noggin using western blot assay. **(C, D)** BMP-2, RUNX2 and osterix were quantified through actin, and p-Smad1/5 was normalized through Smad 1/5/9 **(E, F)** The effect of albiflorin on Wnt canonical pathway-related mRNA expression was measured with or without Wnt/β-catenin inhibitor KYA1797K using RT-PCR assay. **(G, H)** The expression of all mRNA was quantified with respect to that of *Gapdhs*. These results are presented as the mean ± SEM (n = 3 for each experimental). **p < 0.01, *p < 0.05 vs. osteogenic medium cells. ^**††**^ *p* < 0.01, ^**†**^ *p* < 0.05 vs. 100 μg/ml albiflorin-treated cells.

Next, we examined the mechanism by which albiflorin promotes osteoblast differentiation. BMP-2/Smad signaling is an essential signaling pathway that stimulates RUNX2, a key transcriptional marker of osteoblast differentiation. As shown in Figures 6A,C, albiflorin increased the protein expression of BMP-2, RUNX2, and osterix and the phosphorylation of Smad1/5 in a concentration-dependent manner. Noggin is one of the antagonists of BMP-2 and controls the function of BMP-2 in mesenchymal precursor cells (Warren et al., 2003). As shown in Figures 6B,D, it was confirmed that the albiflorin-induced increase in the expression of BMP-2, RUNX2, osterix, and p-Smad1/5 was suppressed by noggin treatment. These results indicate that the effect of albiflorin on osteoblastogenesis is exerted through the promotion of RUNX2 expression via BMP-2/Smad–dependent signaling. Additionally, the expression of Wnt/β-catenin signaling, which was found to play an important role in osteogenesis and fracture repair, was verified at the mRNA level (Xu et al., 2014). Albiflorin increased the mRNA expression of Wnt (*Wnt10b*), β-catenin (*ctnnb1*), LRP5, LRP6, Dvl2, and cyclin D1 (*Ccnd1*) in MC3T3-E1 cells (Figures 6E,G). KYA1797K, known as the Wnt/β-catenin pathway inhibitor, was used to confirm the effect of albiflorin on the gene related to the Wnt/β-catenin mechanism (Figures 6F,H). KYA1797K treatment decreased the expression of Wnt that had been increased by albiflorin, but the difference was not significant, and the expression of β-catenin, LRP5, and Dvl2 was significantly suppressed. However, the expression of LRP6 and cyclin D1 was not affected.

### Albiflorin Enhances the Expression of Osteogenic-Related Genes

**FIGURE 7:**
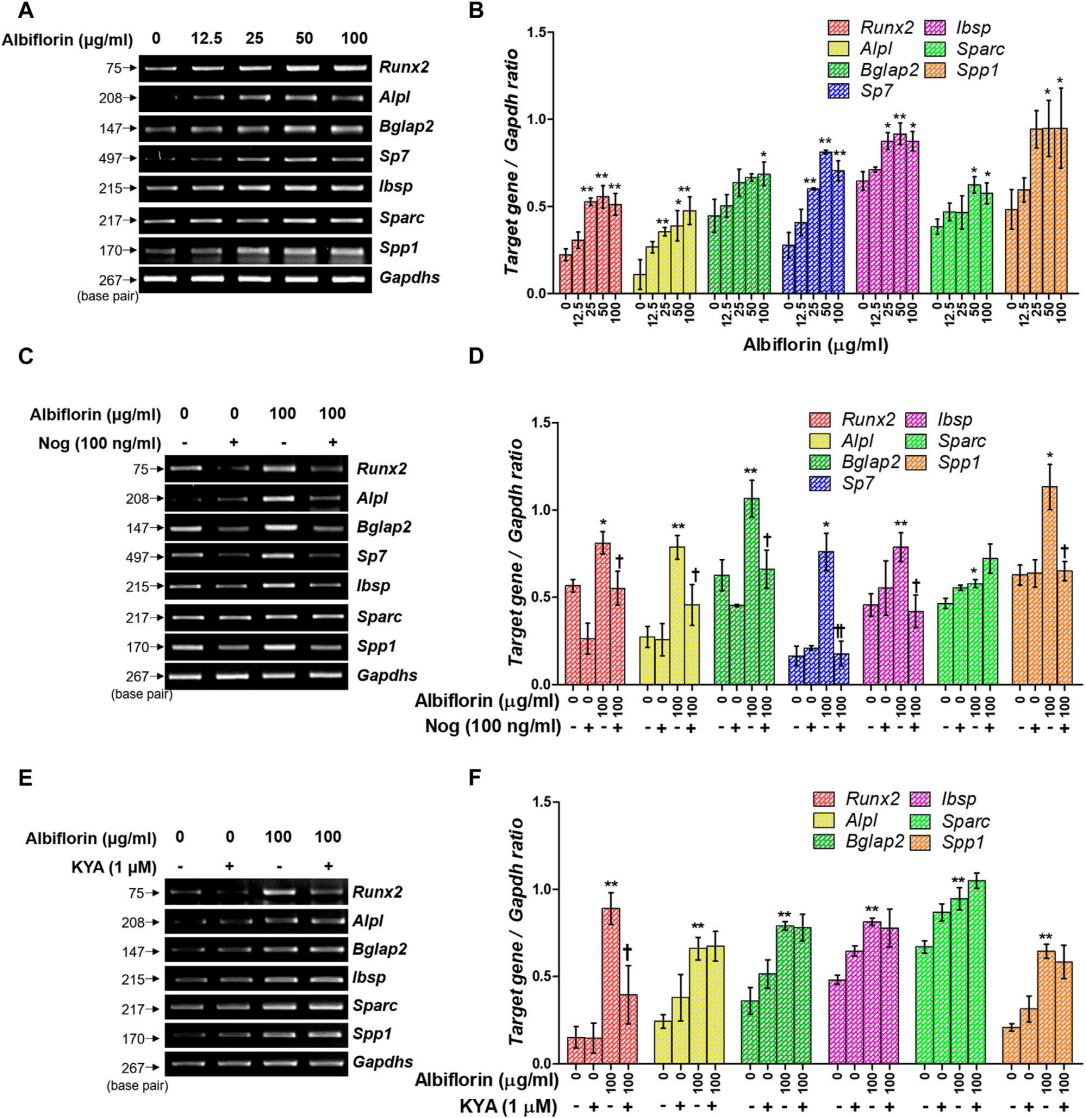
Effect of albiflorin on an osteogenesis-associated gene. **(A)** The *Runx2, Alpl* (ALP)*, Bglap2* (OCN)*, Sp7* (osterix)*, Ibsp, Sparc* (OSN)*,* and *Spp1* (OPN) mRNA expression levels were detected using RT-PCR. **(B)** Each mRNA was quantified with respect to that of *Gapdhs*. **(C)** The BMP-2 inhibitor noggin was used for treatment with or without albiflorin. **(D)** The expression of all mRNA was normalized to that of *Gapdhs*. **(E)** The Wnt/β-catenin inhibitor KYA1797K was used for treatment with or without albiflorin **(F)** The expression of all mRNA was quantified with respect to that of *Gapdhs.* These results are presented as the mean ± SEM (*n* = 3 for each experimental). **p < 0.01, *p < 0.05 vs. osteogenic medium cells. ^††^ *p* < 0.01, ^†^ *p* < 0.05 vs. 100 μg/ml ablbiflorin-treated cells.

Osteoblast differentiation and activation induce the expression of various osteogenic-related genes. As shown in Figures 7A,B, albiflorin enhanced the mRNA expression of ALP (*Alpl*), OCN (*Bglap2*), osterix (*Sp7*), BSP (*Ibsp*), OSN (*Sparc*), and OPN (*Spp1*) in MC3T3-E1 cells. Noggin treatment significantly inhibited the expression of albiflorin-enhanced RUNX2, ALP, OCN, osterix, BSP, and OPN but did not significantly affect the expression of OSN (Figures 7C,D). KYA1797K inhibited the albiflorin-enhanced expression of RUNX2 but did not suppress the expression of bone-related genes, including ALP, OCN, BSP, OSN, and OPN (Figures 7E,F).

### Schematic Diagram of Albiflorin Promoting Osteoblast Differentiation

**FIGURE 8:**
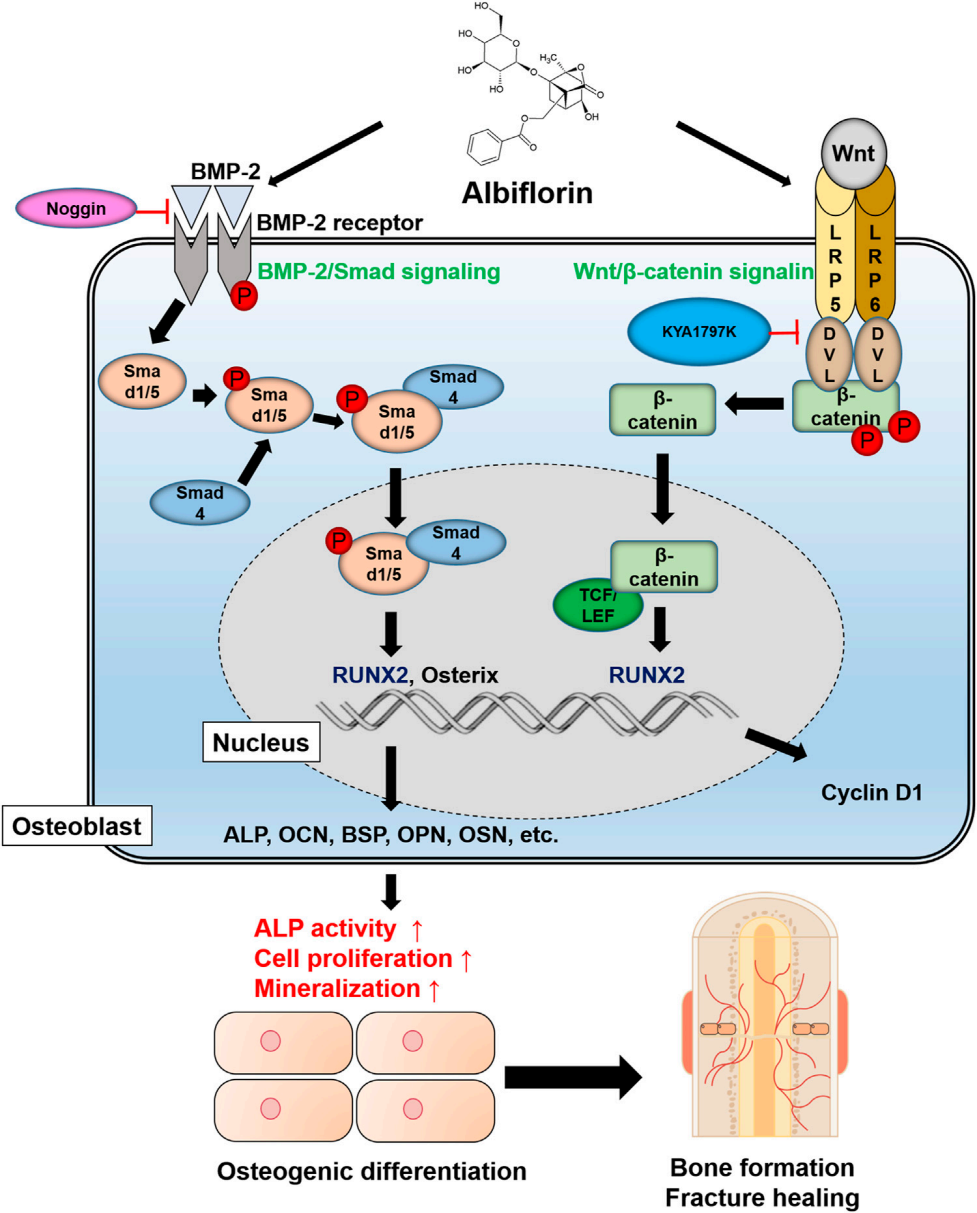
Schematic diagram of albiflorin promoting osteoblast differentiation. Albiflorin stimulates BMP/Smad signaling and Wnt/β-catenin signaling to promote osteoblast differentiation and bone formation.

A schematic diagram of the ostoblastogenesis-promoting effect of albiflorin is shown in Figure 8.

### Albiflorin Promotes Bone Healing in Rat Femoral Fractures

**FIGURE 9:**
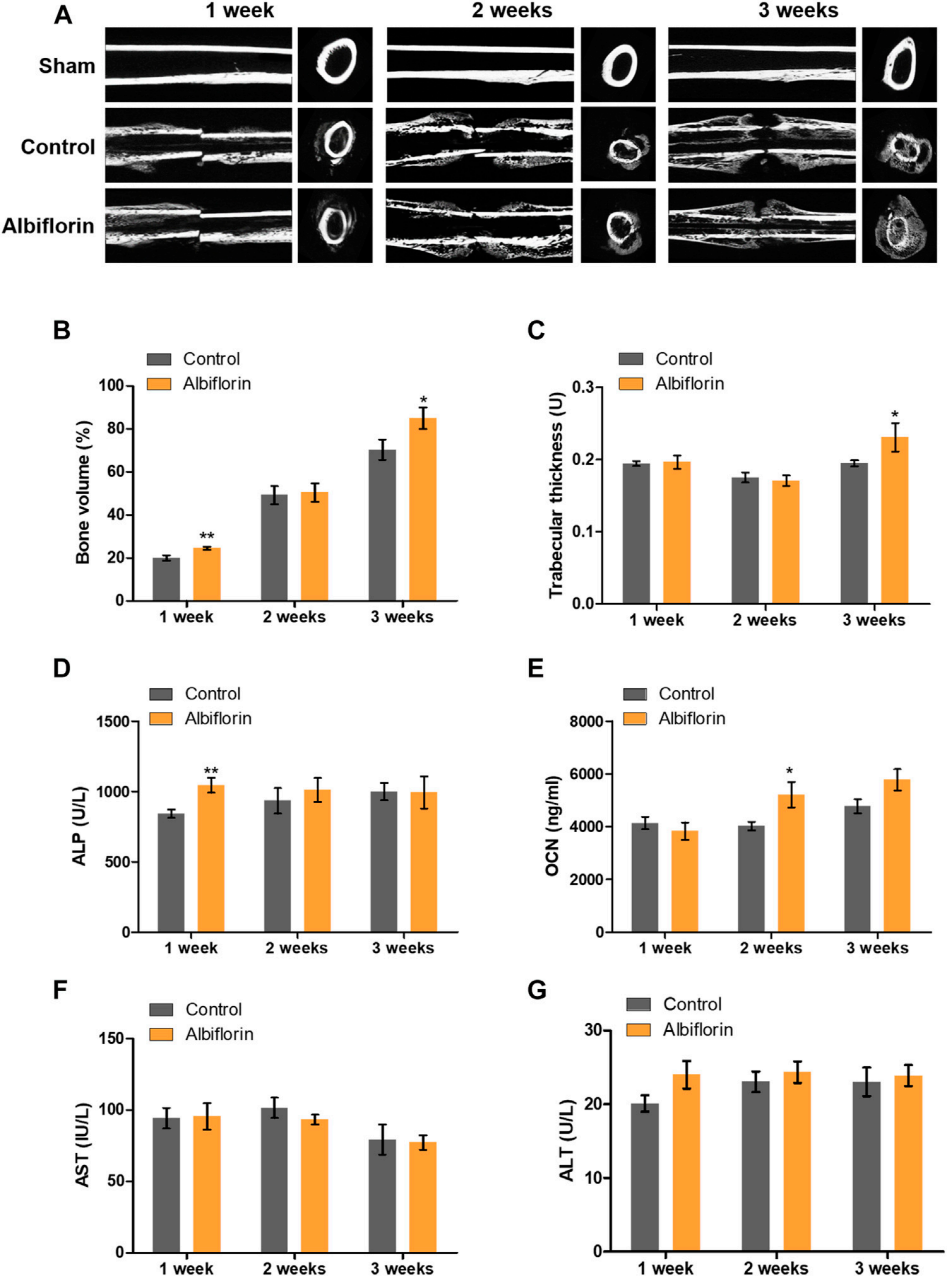
Effect of albiflorin on healing of rat femoral fractures **(A)** Sagittal plane and (left panel) axis plane (right panel) micro-CT image of a fracture-induced femur at 1, 2 and 3 weeks **(B)** Bone volume and **(C)** trabecular thickness were analyzed by micro-CT Expression of **(D)** ALP, **(E)** OCN, **(F)** AST and **(G)** ALT was measured using ELISA in serum. These results are presented as the mean ± SEM (*n* = 6 for each group). *p < 0.05, **p < 0.01 vs. Control.

The fracture union progress with the administration of albiflorin is shown in Figure 9A. After 1 week postfracture, fracture lines were still observed in both the control group and the albiflorin-treated group. After 2 weeks, callus formation was observed, and the fracture line disappeared in the albiflorin-treated group but not in the control group. After 3 weeks, the fracture line still existed in the control group but had disappeared in the albiflorin-treated group. In addition, it was confirmed that the size and density of the formed callus were improved through albiflorin treatment. As shown in Figure 9B, the bone volume with albiflorin treatment was significantly increased compared to that in the control group at 1 and 3 weeks. Trabecular thickness was significantly increased in the albiflorin group at 3 weeks (Figure 9C). After that, bone metabolism index and hepatotoxicity indications in the serum of the albiflorin group were confirmed. As shown in Figure 9D, serum ALP concentrations increased after 1 and 2 weeks of albiflorin treatment. In particular, the albiflorin group showed a very significant increase compared to the fracture group at 1 week. However, there was no significant difference at 3 weeks. The administration of albiflorin significantly increased the expression of OCN in serum compared to the control group at 2 weeks. At week 3, the expression of OCN increased in the albiflorin-treated group, but the difference was not significant (Figure 9E). AST and ALT are representative hepatotoxicity markers in serum, and the administration of albiflorin did not result in a significant difference between the two indicators (Figures 9F,G).

### Albiflorin Improves the Morphological Structure of the Fracture Site

**FIGURE 10:**
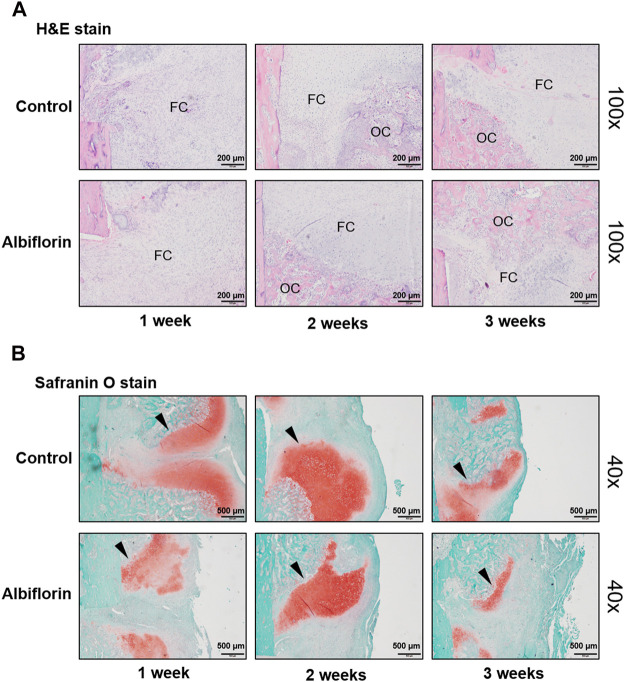
Effect of albiflorin on morphological structure in femoral tissue **(A)** Changes in fibrous and osseous callus at the fracture site were observed by H and E staining (magnification: ×100, scale bar: 200 µm). The abbreviations in the H and E staining are as follows: OC, osseous callus; FC, fibrous callus **(B)** Cartilage areas in the fractured sites were compared by safranin O staining (magnification: ×40, scale bar: 500 µm).

The observation of morphological changes in fracture tissue was verified by H and E staining (Figure 10A). The albiflorin treatment group differed from the control group in fracture recovery and in the proportion of osseous callus to fibrous callus. After 1 or 2 weeks postfracture, the formation of a larger osseous callus was observed in the albiflorin group than in the control group. After 3 weeks, in the albiflorin-treated group, the fibrous callus in the connective tissue at the fracture site was replaced with osseous callus through mature osteoblasts. In addition, we performed safranin O staining to identify fracture lines and cartilage, trabecular bone, cartilage and collagen fibers in tissues (Figure 10B). Consistent with the H and E staining results, the formation of the cartilage area of the albiflorin-treated group was reduced compared to that of the control group after 3 weeks.

### Albiflorin Increases the Expression of Bone Formation Indicators in Femoral Tissue

**FIGURE 11:**
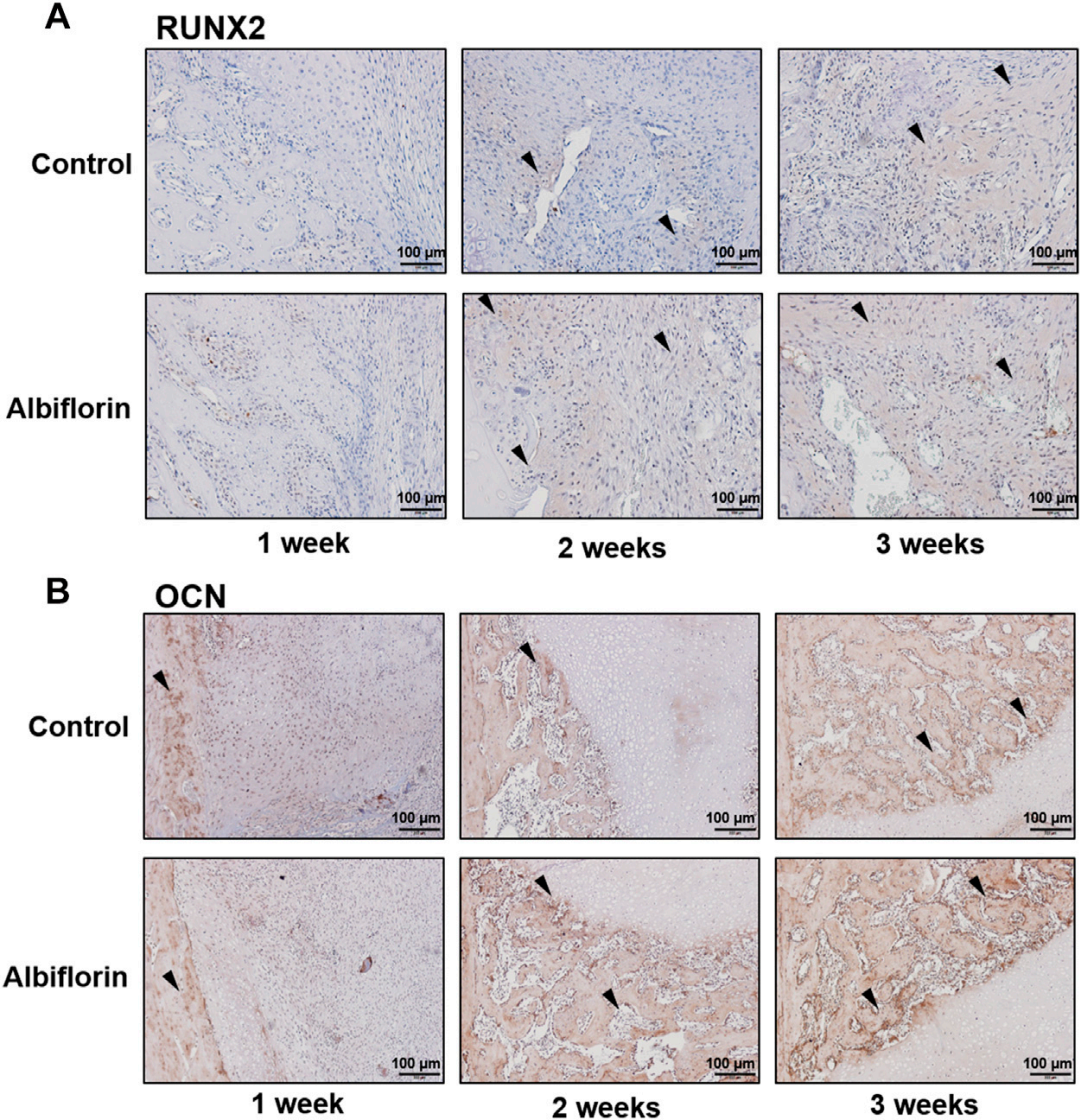
Effect of albiflorin on bone formation indicators in femoral tissue **(A)** Expression of RUNX2 in femoral tissue was analyzed by IHC staining (magnification: ×200, scale bar: 100 µm) **(B)** OCN protein expression at femoral tissue was also measured by IHC staining (magnification: ×200, scale bar: 100 µm).

As shown in Figure 11A, IHC staining was performed to confirm that albiflorin increases the expression of bone formation indicators at the fracture site. After 1 week postfracture, albiflorin did not affect the expression of RUNX2. After 2 or 3 weeks, the albiflorin group had a significantly increased area of RUNX2-positive area in the fracture site. Consistent with the RUNX2 staining results, the expression of the OCN-positive area of the albiflorin-treated group was increased compared to that of the control group each week (Figure 11B). These results indicate that albiflorin promotes early stage recovery by upregulating the expression of RUNX2 in fractured femur tissue.

## Discussion

This study confirmed six new facts: 1) PL induces the expression of BMP-2 and RUNX2 to promote osteoblast differentiation. 2) PL significantly promotes bone union in femoral fractured rats. 3) Both albiflorin and paeoniflorin, known as major components of PL, showed osteoblast-promoting effects, and albiflorin showed better effects. 4) Albiflorin induces osteoblast differentiation through BMP-2/Smad and Wnt/β-catenin signaling. 5) Albiflorin enhances the expression of osteogenic indicators. 6) Finally, it was proven that albiflorin had a positive effect in the early stage of fracture healing.

MC3T3-E1 cells are derived from the bone/calvaria of C57BL/6 mice and have various subclones, including 4, 14, and 24. Among these, subclone four is capable of matrix mineralization, and genetic similarity to primary calvarial osteoblasts has been demonstrated (Hwang and Horton, 2019). Therefore, MC3T3-E1 subclone four is suitable for testing osteoblast traits and has been used in various studies (Kim et al., 2019). For this reason, MC3T3-E1 subclone four was used in this experiment to verify the ability to promote osteoblast differentiation and the mechanisms of albiflorin and PL. Alizarin red S and von Kossa staining verified the formation of mineralization by osteoblasts, which has been demonstrated in previous studies to reflect osteoblast activity (Bills et al., 1974; Gregory et al., 2004). ALP is one of various functional proteins expressed early in the process of mineralization, and ALP staining is commonly used to measure osteoblast differentiation activity (Golub et al., 1992). In this study, both albiflorin and PL increased the formation of mineralization nodules and ALP-positive areas.

BMP-2/Smad signaling is known to be a representative mechanism for promoting osteoblast differentiation. BMP-2 is a member of the transforming growth factor-β (TGF-β) family and plays an important role in osteoblastogenesis and bone formation. Upregulation of BMP-2 expression phosphorylates Smad1/5 with the cooperation of Smad4 in the cytoplasm and finally transfers it into the nucleus (Hay et al., 2001). Thereafter, the differentiation and activity of osteoblasts are induced through increases in RUNX2 and osterix, key transcription factors for osteoblast differentiation (Gilbert et al., 2002). RUNX2 has been demonstrated to be absolutely necessary for embryonic and postnatal skeletogenesis (Takarada et al., 2013), and in addition, Runx2-deficient mice are unable to form bone and show inhibited chondrocyte maturation (Yoshida et al., 2004). Osterix is an osteoblast-specific transcription factor that regulates the expression of various genes during the differentiation of osteoblasts into mature osteoblasts and bone cells. Osterix-mutated embryos do not form bones and do not express osteoblast-specific markers (Sinha and Zhou, 2013). In this study, we confirmed that both PL and albiflorin increased the expression of BMP2, RUNX2 and osterix in a concentration-dependent manner. In addition, it was demonstrated that the ability of albiflorin to promote osteoblast differentiation is mediated by the BMP-2 mechanism through noggin.

The Wnt/β-catenin pathway has been shown in various studies to increase the weight of bone and cortical bone through the activation of mesenchymal stem cells, inhibition of osteoblast death, and induction of osteoblast differentiation. For these reasons, this mechanism is an important strategic indicator of recent bone-related diseases (Baron and Rawadi, 2007; Waalen, 2010; Maeda et al., 2013). The canonical Wnt signaling pathway is associated with the stabilization of β-catenin. In the normal cellular state, the proteasome complex of β-catenin is degraded by glycogen synthase kinase-3β (GSK-3β). When the Wnt mechanism is activated, it is attached to the membrane receptor Frizzled to activate Dvl2 through LRP5/LRP6 coreceptors (Xu et al., 2014). Activated Dvl2 inhibits the interaction of GSK3β with β-catenin, resulting in β-catenin being phosphorylated and then transferred to the nucleus and attached to TCF/LEF, where it upregulates the expression of RUNX2 (Yavropoulou and Yovos, 2007). In this study, it was confirmed that albiflorin enhances the expression of Wnt, β-catenin, LRP5, LRP6, Dvl, and cyclin D1. The experimental results on KYA1797K treatment verified that the ability of albiflorin to enhance osteoblastogenesis is related to the Wnt/β-catenin mechanism. The results of the mechanistic experiment indicate that the osteoblast-promoting ability of albiflorin is exerted through BMP-2/Smad and Wnt/β-catenin signaling.

The promoted osteoblast activity upregulates the expression of various osteogenic genes, such as OCN, BSP, OPN, and OSN. OCN is one of the most abundant proteins in bone and a marker specifically expressed in osteoblasts, and it is generally used as a bone formation marker in serum (Neve et al., 2013). The study by Boskey et al. demonstrated that OCN plays an important role in controlling the maturation of mineralization in OCN-null mice (Boskey et al., 1998). However, it has been recently confirmed that relatively normal bone formation occurs in mice overexpressing OCN (Murshed et al., 2004). For this reason, research on the role of OCN in osteoblasts remains controversial. In this study, albiflorin upregulated the expression of OCN in MC3T3-E1 cells and increased the concentration of OCN in the serum of fractured rats at weeks 2 and 3. BSP is an acidic glycoprotein that develops sufficiently in mineralized tissues. BSP is an early differentiation marker for osteoblasts, and overexpression of BSP increases mature calcium deposition. It also regulates the formation of hydroxyapatite (Gordon et al., 2007). OPN is a bone matrix protein synthesized in osteoblasts. OPN is expressed even in the early stages of osteoblast differentiation but generally in the bone formation process of mature osteoblasts (Sodek et al., 1995). OSN is one of the representative noncollagenous proteins expressed in mineralized tissue. OSN-null bone marrow-derived osteoblasts have impaired osteoblast differentiation and activity and cause mesenchymal precursor cells to be transformed into adipocytes rather than osteoblasts (Delany et al., 2003). In this study, albiflorin induced the expression of various osteogenesis-related genes, such as OCN, BSP, OPN, and OSN, in the early stages of osteoblast differentiation. These results indicate that albiflorin promotes osteoblast activity and bone formation and has shown potential for application in fracture treatment.

The healing process of fractures restores the damaged bone to its original state through the inflammatory phase, reconstruction phase and remodeling phase. During the inflammatory phase, blood from damaged blood vessels at the fracture site forms hematomas, causing an inflammatory reaction that causes acute edema. In the reconstruction phase, fibroblasts are increased, and matrixing occurs due to granulation tissue with angiogenesis. Then, callus and cartilage surrounding the fracture site are formed by osteoblasts, and the ossification process proceeds. Last, in the remodeling phase, the formed bone gradually changes to a mature lamellar bone, while the excess bone produced is absorbed and reformed by osteoclasts (Einhorn and Gerstenfeld, 2015). Since fracture healing proceeds continuously without clear distinctions between each phase, it is important to design the end point for the purpose of the experiment.

In the PL treatment fracture experiment, the experimental period was set to 5 weeks to give sufficient fracture recovery time. As a result, fracture recovery through PL administration was demonstrated by micro-CT, but did not confirm positive results regarding the changes in ALP and OCN in serum. Some previous studies showed sufficient bone formation gene differences at 6 and 8 weeks after fracture, but most studies found that the expression of bone formation genes was confirmed within 4 weeks (Chen et al., 2014; Liu et al., 2016; Yang et al., 2016). Therefore, the reason for the lack of significant difference was considered to be a problem at the time of endpoint of the experiment. For this reason, when evaluating the ability of albiflorin to heal rat femoral fractures, 1, 2, and 3 weeks were designated endpoints of the experiment to examine the fracture recovery process. The micro-CT results showed that the fracture line of the albiflorin-treated group disappeared faster than that of the control group, and it was confirmed that the callus formation rate was accelerated 3 weeks after fracture induction. In histological experiments, albiflorin promoted the change from osseous callus to fibrous callus in the femoral fractured tissue, reduced the area of the cartilage area during the fracture healing phase and upregulated the expression of osteogenic proteins in tissues. In addition, albiflorin increased the concentrations of ALP and OCN in serum at 1 and 2 weeks, respectively. These results indicate that albiflorin promotes fracture healing through upregulation of bone formation markers at the early phase of the fracture. Although no experiment at the end of bone union was conducted, considering the positive effect of albiflorin on the early stage of the fracture process and the effect of promoting osteoblast formation in MC3T3-E1 experiments, it is believed that albiflorin has shown potential as a feasible alternative to promote fracture healing. The results of this study also can be considered to be significant in the following respects. The ability of albiflorin to promote the activity of osteoblasts will be valuable not only for fracture treatment but also for verifying the effectiveness of albiflorin in various bone diseases, including osteoporosis, which occurs when the balance between osteoclasts and osteoblasts is broken (Chen et al., 2018).

The limitations of the results of this study are as follows. 1) Most studies validate only one of the signaling of BMP-2/Smad and Wnt/beta-catenin in the study of osteoblast differentiation activity. In this study, albiflorin showed significant results in activating both mechanisms. However, in the osteogenesis-associated gene verification experiment using KYA1797K, it did not inhibit the increase in gene expression through albiflorin treatment. Further studies are needed to correctly interpret this result, but it is clear that albiflorin has a more positive effect on the BMP-2/Smad signaling. 2) As a result of the study, paeoniflorin along with albiflorin also had an excellent osteoblast differentiation promoting effect. In the future, it is considered that elucidating the combined effect of the two components will also be a goal to improve the quality of research. 3) In addition, PL is composed of ingredients such as gallic acid, (+)−Catechin, methyl gallate, PGG, 6′-O-acetylpaeoniflorin, benzoic acid and paeonol (Bae et al., 2015). Validation of the pharmacological effect of these ingredients will help to understand the fracture healing mechanism of PL and the relatively inconsistent fracture recovery effect of PL compared to albiflorin, so it is worth further research. 4) In this study, we analyzed PL using HPLC-PDA, so the study of mass spectrometry of each component is omitted. Therefore, the analysis of PL using LC–mass spectrometry (MS) is considered to be a method that can improve the quality of the experimental results, so a follow-up study is required.

## Conclusion

In this study, albiflorin, the active ingredient in PL, upregulated BMP-2/Smad and Wnt/β-catenin signaling and induced RUNX2, a key transcription factor for osteoblast differentiation, to express osteogenic genes. Furthermore, it was confirmed that the ability of albiflorin to promote osteoblast differentiation also promotes the ability to repair fractures in the femoral fractured rat model.

## Data Availability

The original contributions presented in the study are included in the article/Supplementary Material, further inquiries can be directed to the corresponding authors.

## Abbreviations

PL, paeonia lactiflora; CCK-8, cell counting kit-8; ALP, alkaline phosphatase; IHC, immunohistochemistry; H and E, hematoxylin and eosin; BMP-2, bone morphogenetic protein 2; RUNX2, runt-related transcription factor 2; OCN, osteocalcin; BSP, bone sialoprotein; OSN, osteonectin; OPN, osteopontin; Wnt10b, protein Wnt-10b (formerly Wnt12); LRP5, low-density lipoprotein receptor-related protein 5; LRP6, low-density lipoprotein receptor-related protein 6; Dvl2, segment polarity protein dishevelled homolog DVL-2; Ccnd1, cyclin D1; PTH, parathyroid hormone; Nog, noggin; KYA, KYA1797K; micro-CT, micro–computed tomography; Tb. Th, trabecular thickness; AST, aspartate transaminase; ALT, alanine transaminase.
